# Mechanisms Behind the Impact of PIWI Proteins on Cancer Cells: Literature Review

**DOI:** 10.3390/ijms252212217

**Published:** 2024-11-14

**Authors:** Piotr Limanówka, Błażej Ochman, Elżbieta Świętochowska

**Affiliations:** Department of Medical and Molecular Biology, Faculty of Medical Sciences in Zabrze, Medical University of Silesia, 19 Jordana, 41-800 Zabrze, Poland; s82955@365.sum.edu.pl (P.L.); d201228@365.sum.edu.pl (B.O.)

**Keywords:** PIWI, signal pathway, cancer

## Abstract

The P-Element-induced wimpy testis (PIWI) group of proteins plays a key role in RNA interference, particularly in the regulation of small non-coding RNAs. However, in recent years, PIWIs have gained attention in several diseases, mainly cancer. Therefore, the aim of this review was to evaluate current knowledge about the impact of PIWI proteins on cancer cells. PIWIs alter a number of pathways within cells, resulting in significant changes in cell behavior. Basic processes of cancer cells have been shown to be altered by either overexpression or inhibition of PIWIs. Regulation of apoptosis, metastasis, invasion, or proliferation of cancerous cells by these proteins proves their involvement in the progression of the malignancy. It has been revealed that PIWIs are also connected with cancer stem cells (CSCs), which proves their ability to become a therapeutic target. However, research on this topic is still fairly limited, and with significant differences between cancer types, it is necessary to refrain from making any decisive conclusions.

## 1. Introduction

P-Element-induced wimpy testis (PIWI) proteins belong to the highly conserved Argonaute family, primarily implicated in RNA interference mechanisms [1]. Since their characterization in 1997, PIWI proteins have been recognized as essential in the division process of germline stem cells (GSCs) [2], particularly in enabling self-renewal division of GSCs [3]. Structurally, PIWI proteins contain two RNA-binding domains: the N-terminal PAZ domain region, comprising approximately 110 amino acids, and the C-terminal 300-amino-acid Piwi domain [4]. There are four known human PIWI homologs: PIWIL1 (HIWI), PIWIL2 (HILI), PIWIL4 (HIWI2), and PIWIL3 (HIWI3), all of which have been shown to be expressed in a number of different cancer tissues [5]. The PIWIL1 protein is encoded by the *HIWI* gene, which is located at the long arm of chromosome 12, band 12q24.33. This region produces a 3.6 kb mRNA that can be translated into a highly basic protein consisting of 852 amino acids, with a molecular weight (Mw) of 96.56 kDa. The PIWIL2 protein, encoded by the *HILI* (*PIWIL2*) gene on chromosome 8, comprises 973 amino acids and has a Mw of around 109.85 kDa. The *HIWI3* (*PIWIL3*) gene, located on chromosome 22, encodes the PIWIL3 protein, which is 882 amino acids in length and has a Mw of 101.12 kDa. The PIWIL4 protein, encoded by the *HIWI2* (*PIWIL4*) gene on chromosome 11, consists of 852 amino acids, with an approximate Mw of 96.59 kDa [6,7].

PIWI proteins bind to a class of small non-coding RNAs known as piwi-interacting RNAs (piRNAs) [8]. These piRNAs, typically 26–31 nucleotides in length, serve as epigenetic regulators and are integral in gene expression regulation within the germline cells, with notable implications for male fertility [9,10].

The functions of PIWI proteins were originally described in the context of GSC division. However, subsequent research has revealed a broader range of PIWI functions across cellular processes. PIWI has been shown to induce transcriptional repression by establishing a repressive chromatin state after the identification of a target that is complementary to the associated piRNA [11]. Mutation in the *piwi* gene has been shown to alter the posttranscriptional gene silencing and transcriptional gene silencing of the alcohol dehydrogenase transcription unit [12]. Piwi also contributes to heterochromatic H3K9 trimethylation marks on transposons and nearby genomic regions, with the HMG protein Maelstrom playing a critical role in PIWI-mediated silencing [13]. Additionally, PIWI promotes the euchromatic state of telomere-associated sequences on chromosome 3’s right arm, a process integral to GSC maintenance and mediated by piRNA interactions [14]. Beyond these roles, PIWI proteins are involved in mRNA decay and enhance transcription factor activity, which can impact cellular signaling pathways [15,16]. The interaction of piRNAs with PIWI proteins forms piRNA-induced silencing complexes (piRISCs), which have distinct roles depending on cellular localization. In mitochondria, piRISCs are essential for piRNA biogenesis by processing piRNA precursors, achieved via Zucchini (Zuc)-mediated cleavage. The primary function of piRISCs in the nucleus is the silencing of transposons. This is achieved either through DNA methylation in transposon-associated regions or by modulating the chromatin condensation process around transposon sequences [17,18,19]. Loss of transposon silencing by piRISCs can compromise genome stability, as unchecked transposon activity may lead to mutations in genes responsible for cell cycle regulation and DNA repair, increasing the risk of oncogenic transformation. This suggests that PIWI proteins may represent a promising research target in the study of cancer pathogenesis [20,21,22,23]. Another class of proteins that plays a critical role in the regulation of piRNA biogenesis, the stabilization of piRISC complexes, and the maintenance of genome integrity through their interaction with PIWI proteins are the Tudor domain-containing proteins (TDRD, Tudor Domain Proteins) [24,25]. TDRD proteins, characterized by the presence of multiple Tudor domains, are highly conserved across diverse species and are prominently expressed during key developmental stages in germline cells [26,27]. TDRD proteins interact with PIWI proteins through specific binding between the extended Tudor (eTudor or eTud) domains of TDRDs and the RG/RA repeat motifs located at the N-terminal regions of PIWIs. The symmetric dimethylation of arginine residues within these RG/RA repeats is considered crucial for facilitating interaction with eTudor domains [28,29,30,31]. PIWI proteins are recognized by TDRDs in an arginine methylation-dependent manner. The N-termini of PIWI proteins contain multiple arginine methylation sites, and it has been shown that human TDRD11 and Drosophila melanogaster Tudor proteins utilize their eTudor domains to specifically recognize symmetrically dimethylated arginine residues on the N-termini of their respective PIWI protein partners [32,33]. Given the role of TDRD proteins in interacting with PIWI proteins and their participation in key biological processes, such as histone modification and the DNA damage response, mutations and overexpression of TDRD-related genes have been linked to cancer development [34,35,36,37]. PIWI expression has also been shown to be connected with the hypothalamic–pituitary–gonadal (HPG) axis [38,39]. This hormonal regulation aligns with PIWIs’ involvement in gonadal development and germline maintenance. Other mechanisms influencing PIWI expression include environmental stressors; for instance, Mikhaleva et al. observed a shift in PIWI localization from the nucleoplasm to the nucleolus in response to heat shock [40]. PIWIL2 expression varies with keratinocyte differentiation states [41], and hypoxic postconditioning has been found to downregulate this protein’s expression [42]. Notably, research by Kitamura et al. identified sulfonamides capable of binding to the PAZ domain of PIWI proteins, thus inhibiting RNA interference activity [43]. Despite these insights, regulatory mechanisms governing PIWI protein expression remain incompletely understood.

Altered piRNA expression has been associated with various diseases, including neurodegenerative disorders [44], cardiovascular diseases, ref. [45], and others. In cardiovascular diseases, serum piRNAs have shown promise as biomarkers, especially in pulmonary hypertension [45]. piRNAs have also emerged as promising biomarkers or therapeutic targets in cancer research [46,47,48]. Their role is also shown to be connected with processes such as differentiation, remodeling, and regeneration of myocytes [45,49]. The dysregulation of PIWI protein expression has been observed in various processes involved in disease development. PIWIL2 and PIWIL4 may play roles in pancreatic beta cell function, contributing to development of type 2 diabetes [50]. PIWIL2 and PIWIL4 have also been implicated in the unfolded protein response in human airway epithelial cells [51], with PIWIL2 potentially upregulated by NF-E2-related factor 2, contributing to the attenuation of radiation-induced lung fibrosis [52].

In cancer, PIWI protein expression is frequently altered. PIWIL4 has been detected at elevated levels in MDA-MB-231 cells, a breast cancer cell line [53], and PIWIL2 has been proposed as a promising biomarker for breast cancer, being expressed across various disease stages and present in all examined tissue microarray cores [54]. PIWIL1 has been proposed as a progression marker for both cervical squamous cell carcinoma and glioma, with expression levels correlated to tumor grades in these cancers [55,56]. Elevated PIWI protein expression has also been reported in gastric cancer [57,58]. Animal studies have shown that modulating PIWI expression can influence tumor growth, raising the possibility of targeting PIWI proteins as a therapeutic strategy [59,60,61,62,63,64,65,66,67].

A number of studies have shown how PIWI proteins can alter processes within cells, resulting in a number of changes, ultimately also carcinogenesis. Therefore, the aim of this review was to explore the mechanisms by which PIWI proteins influence cancer cell behavior. In this review, we present and synthesize the most significant advancements in the study of the role of PIWI protein family members in the pathogenesis of cancer and various other diseases. We describe the established regulatory interactions and discuss key issues related to the mechanisms of action and interactions of PIWI proteins within target cells.

## 2. Methodology

The articles analyzed in this narrative review included experimental studies in human cell cultures, focusing on the functions of PIWI proteins in oncogenic processes and processes associated with tumor progression, conducted in various human neoplastic cell cultures. Selection criteria for the PIWI protein-related articles encompassed studies retrieved from the PubMed database using the following keywords and Boolean operators, sourced from the MESH database: Argonaute Proteins and cancer; Argonaute Proteins and neoplasm; Argonaute Proteins and carcinogenesis; Argonaute Proteins and Cancer Stem Cells; Argonaute Proteins and Neoplastic Stem Cell. Keywords were further refined for each specific PIWI protein studied, and included the following: PIWIL1 or PIWIL2 or PIWIL3 or PIWIL4 and cancer; PIWIL1 or PIWIL2 or PIWIL3 or PIWIL4 and neoplasm; PIWIL1 or PIWIL2 or PIWIL3 or PIWIL4 and carcinogenesis; PIWIL1 or PIWIL2 or PIWIL3 or PIWIL4 and Cancer Stem Cells; PIWIL1 or PIWIL2 or PIWIL3 or PIWIL4 and Neoplastic Stem Cell. To ensure the high quality of the review, preference was given to peer-reviewed articles. Preliminary selection was conducted by screening titles and abstracts, followed by a full-text analysis of articles selected for inclusion. The authors independently described selected issues and subsequently cross-verified each other’s study interpretation to confirm that the study descriptions accurately reflected the source material. The presented figures were generated based on the integration of the results of the discussed research articles, described in detail in the main part of the review, using Canva.

## 3. Cell Cycle, Proliferation and Apoptosis

One of the most well-described mechanisms of PIWI-meditated tumorigenesis is their involvement in regulation of the cell cycle, proliferation, and apoptosis.

### 3.1. PIWIL1

**Figure 1:**
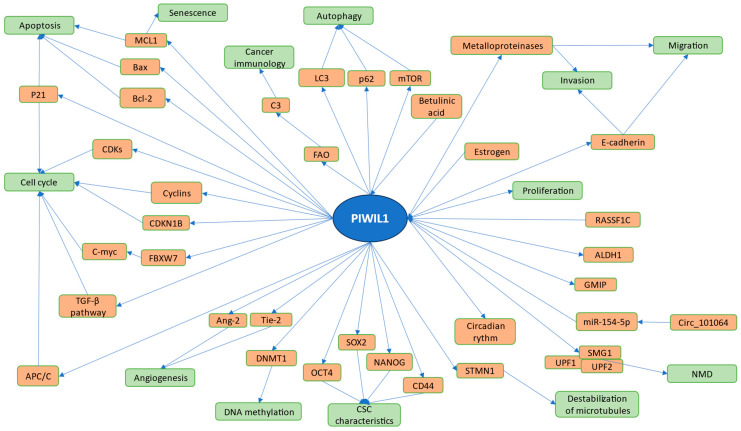
Pathways of PIWIL1 in cancer cells. Processes regulated by PIWI proteins and their mechanisms are included in the figure. PIWIL1 can influence the cell cycle, angiogenesis, DNA methylation, CSC characteristics, destabilization of microtubules, NMD, proliferation, invasion, migration, autophagy, cancer immunology, senescence, and apoptosis of cancer cells. This is achieved through a number of different mechanisms, including altered expression of CDKs, cyclins, angiogenesis-associated factors, transcription factors connected with stemness, and pathways such as TGF-β. Worth noting is the ability of betulinic acid, estrogen, RASSF1C, and non-coding RNAs to influence activity of PIWL1. The following are featured in the figure: Fatty Acid Oxidation (FAO), Cyclin-dependent kinase inhibitor 1B (CDKN1B), F-box and WD repeat domain containing 7 (FBXW7), Myeloid cell leukemia-1 (MCL1), Cyclin-dependent kinase (CDK), Anaphase-promoting complex (APC/C), B-cell lymphoma 2 (Bcl-2), BCL2 associated X (Bax), Transforming growth factor beta (TGF-β), Angiopoietin 2 (Ang-2), Angiopoietin-1 receptor (Tie-2), DNA methyltransferase 1 (DNMT1), Complement component 3 (C3), Microtubule-Associated Protein 1 Light Chain 3 (LC3), Mammalian target of rapamycin (mTOR), (Octamer-binding transcription factor 4) OCT-4, SRY (sex-determining region Y)-box 2 (SOX2), Nanog homeobox (NANOG), Stathmin 1 (STMN1), GEM interacting protein (GMIP), Aldehyde Dehydrogenase 1 (ALDH1), and Ras-Association Domain Family 1C Protein (RASSF1C).

A number of studies have shown possible regulation of the cell cycle by PIWI proteins. Huang et al. reported a possible influence of PIWIL1 on glioma stem-like cells, where it regulates G1/S progression, possibly through changing the stability of mRNA of CDKN1B, CCND2, and FBXW7, leading to reduced expression of c-Myc. Silencing of PIWIL1 also resulted in higher expression of MCL1 and induced the senescence phase in cells [67]. Yang et al. reported the possible use of betulinic acid as an anticancer agent, because of its effect on gastric cancer cells, which was growth inhibition, induction of apoptosis, and G2/M stage cell cycle arrest. It appears that this phenomenon is achieved through the PIWIL1/Cyclin B pathway, where betulinic acid reduces the expression of both PIWIL1 and Cyclin B [68]. PIWIL1 was also shown to be responsible for the progression of breast cancer both in vivo and in vitro. It might be connected to the increased expression of TβRI, TβRII, CDK4, CDK6, and CDK8. Additionally, inhibition of PIWIL1 arrests cells at the G2/M stage [69]. Decreased activity of PIWIL1 in glioma cells led to suppressed cell proliferation, increased apoptosis, altered expression of p21, cell cycle arrest at G0/G1, and increased and decreased expression of Bcl-2 and Bax, respectively [70]. Li et al. described the mechanism of PIWIL1-induced upregulation of STMN1 expression at the protein level. It is achieved by suppressing ubiquitination and degradation of STMN1, which is conducted by RLIM. PIWIL1 can bind to STMN1 and tubulin, forming a PIWIL1/STMN1/tubulin complex. Additionally, PIWIL1 represses STMN1 phosphorylation at Ser16, which is meditated by CaMKII. It results in the destabilization of microtubules and subsequent increase in proliferation of HeLa and HepG2 cells [71]. PIWIL1 has been also examined in renal cell proliferation. The effects of PIWIL1 depletion on apoptosis were observed. Flow cytometry analysis revealed no significant changes in the number of apoptotic or necrotic cells compared to non-depleted controls. However, a marked decrease in cell proliferation was observed, as demonstrated by a wound healing assay and cell viability analysis. The study concludes that PIWIL1 is essential for promoting the proper proliferation of renal cells and its depletion significantly impacts cellular growth rates without affecting apoptosis. This process may be implicated in renal cancer pathogenesis [22]. Yang et al. revealed results showing that overexpression of PIWIL1 promoted proliferation of Caro-2 and HT-29 cells through increased global DNA methylation [72]. Research conducted by Siddiqi et al. investigated a similar mechanism of DNA methylation of tumor-suppressing genes, translating this into the tumorigenic properties of cancer cells. Furthermore, it was revealed that such an effect can be suppressed by DNA-methyltransferase inhibitors [73]. Inhibition of the proliferation and colony-forming ability of K562 cells was achieved through overexpression of PIWIL1. Decreased Bcl-2 expression; increased Bax expression; and activated caspase-3, caspase-9, and cleaved PARP were also observed to be effects of PIWIL1 [74]. In another study on K562 cells, the authors detected an association between increased levels of PIWIL1 and apoptosis [60]. PIWIL1 has also been found to be a critical factor in cervical cancer development by Kunnummal et al. In this study, PIWIL1 was shown to be highly expressed in cervical cancer cells, with a positive correlation to HPV oncogene levels. Knockout of PIWIL1 in CaSki cells, achieved via CRISPR-Cas9, led to a significant reduction in cell viability, increased apoptosis, and decreased proliferation, confirming PIWIL1’s role in promoting cancer cell growth and stemness. The study found that PIWIL1 could serve as a potential therapeutic target for inhibiting tumor growth and invasion in cervical cancer [75]. Worth mentioning is study by Wang et al. where upregulation or downregulation of PIWIL1 did not result in a change in apoptosis in MM cells. However, other mechanisms such as mitophagy and autophagy appeared to be altered by PIWIL1. It was suggested that PIWIL1 achieves this through modulating the autophagy-related proteins LC3, p62, and mTOR and the involvement of PINK1/PARKIN pathway [76]. Wang et al. revealed how miR-154-5p inhibits cell proliferation and metastasis in glioma through direct targeting of PIWIL1′ 3′UTR [77]. Furthermore, Zhou et al. introduced the Circ_101064/miR-154–5p/PIWIL1 axis, adding that Circ_101064 knockdown downregulates expression of PIWIL1, resulting in suppressed proliferation [78]. PIWIL1 knockdown in lung cancer cells inhibits proliferation, promotes apoptosis, and reduces the number of ALDH-1-positive cells [64]. Chen et al. presented results indicating that estrogen could upregulate the expression of PIWIL1 in ERα-positive endometrial cancer cells and downregulate the expression of PIWIL1 in the ERα-negative endometrial cancer cell line. The authors suggest that half-ERE is necessary for the binding of the ERα onto the PIWIL1 promoter; however, they do not rule out the possibility that some yet-unidentified ERα-associated proteins take part in this process. These results showed the possible mediating effect of PIWIL1 on E2-stimulated endometrial carcinogenesis [79]. Shi et al. reported a piRNA-independent PIWIL1 mechanism of nonsense-mediated mRNA decay (NMD) in gastric cancer cells. PIWIL1 forms a complex with UPF1, UPF2, and SMG1, which creates NMD machinery that degrades mRNAs and lncRNAs, supposedly resulting in tumor growth [66]. Inhibition of RASSF1C results in reduced expression of PIWIL1. It was suggested that this happens through the activation of the MEK-ERK1/2 pathway. Such a correlation implies the involvement of these proteins in the initiation of early lung cancer and its progression [80]. Amaar and Reeves reported the possible role of the RASSF1C-PIWIL1-piRNA pathway in the methylation of genes such as *GMIP*, which appears to be a possible lung cancer biomarker [81]. PIWIL1 was revealed to be responsible for the regulation of the *PTEN* gene in endometrial cancer. This PIWI protein could regulate the expression of DNA methyltransferase 1, which would eventually result in hypermethylation of *PTEN*. The authors suggest that the PIWIL1/DNMT1/PTEN signaling axis may play an important role in the progression of type I endometrial cancer [82]. Mechanisms behind PIWIL1′s impact on carcinogenesis are summarized in Figure 1. It is worth noting that a significant number of studies have shown different ways to regulate cell division and apoptosis of cancer cells by PIWIL1. Nevertheless, it is observed to also have other properties, such as involvement in cancer stem cell (CSC) characteristics, angiogenesis, DNA methylation, and cancer immunology.

### 3.2. PIWIL2

**Figure 2:**
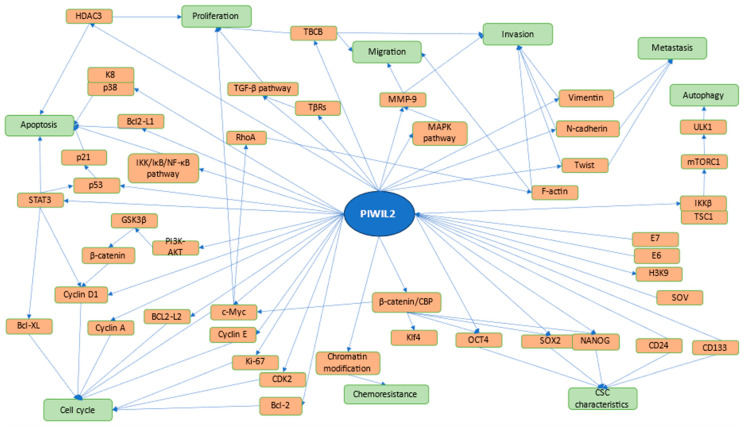
Pathways of PIWIL2 in cancer cells. Processes regulated by PIWI proteins and their mechanisms are included in the figure. PIWIL2 is reported to be involved in regulation of proliferation, apoptosis, cell cycle, chemoresistance, CSC characteristics, autophagy, and metastatic properties. A number of different mechanisms are involved in this regulation, such as PI3K-AKT, MAPK, TGF-β pathways, chromatin modification, and regulation of STAT3. Moreover, E6 and E7 proteins and SOV are able to alter expression of PIWIL2. The following are featured in the figure: Histone deacetylase 3 (HDAC3), Keratin 8 (K8), Bcl-2-like protein 1 (Bcl2-L1), Signal transducer and activator of transcription 3 (STAT3), Phosphoinositide-3-kinase (PI3K), Protein kinase B (AKT), Glycogen synthase kinase-3 beta (GSK3β), B-cell lymphoma-extra large (Bcl-xL), Bcl-2-like protein 2 (Bcl2-L2), Transforming growth factor beta (TGF-β), Nuclear factor kappa-light-chain-enhancer of activated B cells (NF-κB), Ras homolog family member A (RhoA), Antigen Kiel 67 (Ki-67), Cyclin-dependent kinase 2 (CDK2), B-cell lymphoma 2 (Bcl-2), Transforming growth factor beta receptor (TβR), Tubulin-folding cofactor B (TBCB), Mitogen-activated protein kinase (MAPK), Matrix metallopeptidase 9 (MMP-9), Krüppel-like factor 4 (KLF4), (Octamer-binding transcription factor 4) OCT-4, SRY (sex-determining region Y)-box 2 (SOX2), Nanog homeobox (NANOG), Sodium orthovanadate (SOV), Histone H3 lysine 9 (H3K9), inhibitor of nuclear factor kappa-B kinase subunit beta (IKKβ), mammalian target of rapamycin complex 1 (mTORC1), and unc-51-like autophagy activating kinase 1 (ULK1).

Another study also showed how overexpression of PIWIL2 may result in the upregulation of a number of genes, such as cyclin D1, STAT3, BCL2-L1, BCL2-L2, and Ki-67, which are known for their importance in cell cycle regulation. It was suggested that PIWIL2 accelerated cellular division and inhibited apoptosis through interaction in the STAT3/BCL-XL and cyclin D1 pathways [83]. PIWIL2 is also involved in the β-catenin pathway, where it can upregulate the expression of β-catenin and its downstream target gene, CyclinD1. This change is attributed to the activation of the PI3K-AKT pathway and phosphorylation of GSK3β, and thereafter the suppression of the phosphorylation and degradation of β-catenin induced by GSK3β. It results in the accumulation of β-catenin in the nucleus, eventually resulting in activation and transcription of CyclinD1 [84]. Qu et al. observed that in non-small cell lung cancer cells, PIWIL2 overexpression was responsible for proliferation, while suppression arrested cells at the G2/M stage. Moreover, it was shown that the expression of CDK2 and Cyclin A corelated with expression of PIWIL2 [63]. In cervical cancer cells, HPV16 infection might increase the expression of PIWIL2. It would result in altered proliferation and migration of these cells, possibly through altered expression of cyclin D1 and cyclin E by PIWIL2 [85]. Lee et al. discovered a mechanism through which PIWIL2 can be involved in apoptosis and proliferation. Supposedly, this protein can alter pathways such as STAT3/Bcl-XL and STAT3/cyclin D1, which are responsible for apoptosis and proliferation, respectively [86,87]. In SW480 cells, PIWIL2 appears to have an anti-apoptotic effect by upregulating STAT3 and Bcl2-L1. Furthermore, increased expression of Cyclin D1 and Ki-67 was also observed [88]. Research based on HeLa cells revealed a possible complex formed by PIWIL2, STAT3, and c-Src. STAT3 can be phosphorylated by c-Src, which, after translocation to the nucleus, can suppress expression of p53. This phosphorylation was significantly increased in PIWIL2-overexpressed cells [89]. PIWIL2 can inhibit the degradation of HDAC3 meditated by Siah2 and increase the activity of HDAC3 by promoting interaction between HDAC3 and CK2α. Inhibition of PIWIL2 leads to increased expression of p53 and its downstream protein, p21. These changes promote proliferation and suppress apoptosis of cancer cells [90]. In HepG2 cells, PIWIL2 can impact TGF-β signaling, inducing proliferation. PIWIL2 competes with TβRs for Hsp90, decreasing the stability of TβRs, subsequently increasing its degradation [91]. Another study showed how PIWIL2 can increase the stability of keratin 8 (K8). It was revealed that the PIWIL2/K8/p38 complex can be formed, resulting in an increased phosphorylation level of K8, therefore suppressing ubiquitin-mediated degradation of K8. Considering K8’s role in Fas-mediated apoptosis, it appears that PIWIL2 plays an important role in tumorigenesis [92]. Another research paper showed how PIWIL2 can destabilize microtubules. This phenomenon is meditated by PIWIL2, inhibiting Gigaxonin-mediated Tubulin cofactor B (TBCB) ubiquitination. Furthermore, PIWIL2 can promote interaction of HSP90 with TBCB and suppress interaction of Gigaxonin with TBCB. Lastly, it was revealed that PIWIL2 can inhibit PAK1-mediated phosphorylation of TBCB. These changes result in altered proliferation [93]. Another piece of research showed how PIWIL2 impacts the expression of c-Myc, which can alter cell cycle and proliferation. It was reported that this phenomenon is possible through a mechanism where PIWIL2 promotes NME2 binding to the G4-motif within the c-Myc promoter [94]. In esophageal squamous cell carcinoma cell lines, overexpression of PIWIL2 increased proliferation, suppressed apoptosis, and induced autophagy. Furthermore, this inhibition of apoptosis might be mediated by the IKK/IκB/NF-κB pathway, which can be activated by PIWIL2. Autophagy can be altered by PIWIL2, through competitive inhibition of the binding of IKKβ to TSC1, resulting in the deactivation of mTORC1, which suppress ULK1 phosphorylation, subsequently leading to autophagy [95]. The role of PIWIL2 in promoting tumorigenesis in colorectal cancer during α2-adrenoceptor agonist dexmedetomidine (DEX) exposure has been investigated by Dong et al. The study demonstrated that DEX exposure upregulates PIWIL2 expression, particularly in CRC cell lines, contributing to, among others, increased cell proliferation. Furthermore, the study elucidates the role of the PIWIL2/Siah2/HIF1α signaling pathway in DEX-induced tumorigenesis. In vivo experiments using mouse xenograft models supported the in vitro findings, showing that DEX-induced tumor growth and metastasis are mediated by PIWIL2 signaling [96]. Tian et al. reported the impact of sodium orthovanadate (SOV) on SH-SY5Y cells. SOV induced an increase in the number of cells in the G2/M and S phases of the cell cycle. Furthermore, SOV inhibited expression of PIWIL2 mRNA and the protein itself [97]. Feng et al. observed that HPV proteins E6 and E7 can reactivate PIWIL2, which can subsequently result in increased proliferation. Overexpression of PIWIL2 or activation of E6 and E7 results in H3K9 acetylation but suppressed H3K9 trimethylation. These changes can induce epigenetic changes within cells, contributing to the possible transformation of cervical epithelial cells into tumor-initiating cells (TICs) [98]. PIWIL2 has also been found to be involved in the effects of the overexpression of piR-4447944 in prostate cancer cells. Peng et al. discovered that piR-4447944 binds to PIWIL2, forming a piR-4447944/PIWIL2 complex, which inhibits the tumor suppressor gene *NEFH* at the posttranscriptional level. The study demonstrated that piR-4447944 plays a critical role in promoting androgen-independent growth in prostate cancer by interacting with PIWIL2 [99]. The involvement of PIWIL2 in tumorigenesis is outlined in Figure 2. PIWIL2 appears to be involved in many diverse mechanisms, as presented in the figure. The most prominent involvement of PIWIL2 can be seen in CSC characteristics and cell division control. However, an association with chemoresistance and autophagy is worth noting.

### 3.3. PIWIL3

In gastric cancer cells, inhibition of PIWIL3 was able to arrest cells in the G0/G1 phase [100]. Another research paper suggested that PIWIL3 is the target of enoxacin. This implies that re-expression of PIWIL3 in cancer cells results in repressed RNAi, subsequently promoting cancer cell growth [101].

### 3.4. PIWIL4

**Figure 3:**
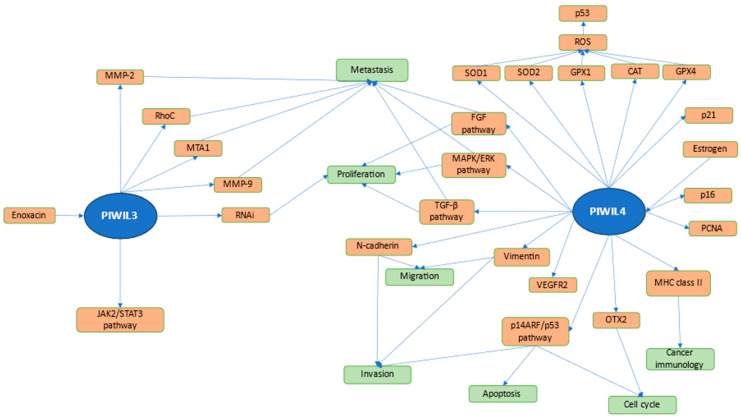
Pathways of PIWIL3 and PIWIL4 in cancer cells. Processes regulated by PIWI proteins and their mechanisms are included in the figure. Both PIWIL3 and PIWIL4 are associated with proliferation and metastasis. Furthermore, PIWIL4 can impact migration, invasion, apoptosis, cell cycle, and cancer immunology. PIWIL3 can influence cancer cells through regulation of the JAK2/STAT3 pathway, and expression of metalloproteinases, RNAi, and others. PIWIL4 can regulate important pathways in cancer biology, such as TGF-β, MAPK, and FGF. It is worth noting that estrogen can impact PIWIL4, while Enoxacin can impact PIWIL3. The following are featured in the figure: Matrix metalloproteinase-2 (MMP-2), ras homolog family member C (RhoC), Metastasis-associated protein (MTA1), Matrix metallopeptidase 9 (MMP-9), RNA interference (RNAi), Transforming growth factor beta (TGF-β), Mitogen-activated protein kinase (MAPK), extracellular signal-regulated kinase (ERK), Fibroblast growth factor (FGF), Vascular endothelial growth factor receptor 2 (VEGFR2), Orthodenticle homeobox 2 (OTX2), Proliferating cell nuclear antigen (PCNA), Superoxide dismutase 1 (SOD1), Superoxide dismutase 2 (SOD2), Glutathione peroxidase 1 (GPX1), Catalase (CAT), Glutathione Peroxidase 4 (GPX4), and Reactive oxygen species (ROS).

In cervical cancer cells, PIWIL4 presents oncogenic characteristics. It was suggested that through altering the p14ARF/p53 pathway, PIWIL4 was able to regulate invasion, cell cycle, and induce apoptosis [102]. Das et al. reported the possible influence of PIWIL4 on DNA damage in fibrosarcoma cells. PIWIL4 overexpression was associated with increased phosphorylation of H2AX, cell cycle arrest in the G2/M phase, apoptosis, and elevated concentration of ROS. Furthermore, PIWIL4 was found to increase expression of p53, p21, and caspase-3. Intracellular ROS accumulation was caused by decreased expression of SOD1, SOD2, GPX1, GPX4, and CAT. These findings suggest that increased p53 expression is the result of the accumulation of ROS caused by PIWIL4 [103]. In MDA-MB-231 cells derived from triple-negative breast cancer, PIWIL4 was reported to be able to upregulate the TGF-β, MAPK/ERK, and FGF pathways, which resulted in increased migration, proliferation, and survival of cancer cells [53]. Sivagurunathan et al. reported the involvement of PIWIL4 in retinoblastoma cells. PIWIL4 knockdown suppressed expression of OTX2 and VEGFR2. OTX2 inhibition was suggested to be the reason for cell cycle arrest. Furthermore, the expression of PCNA and p16 was downregulated in PIWIL4 knockdown Y79 cells [104]. The pathways associated with PIWI3 and PIWI4 are summarized in Figure 3. The number of studies concerning regulation of different pathways by PIWIL3 and PIWIL4 is markedly lower than that of PIWIL1 and PIWIL2, which can be seen in the figure. PIWIL3 is associated with metastasis and proliferation, while PIWIL4 is also observed to have an impact on other processes such as cell cycle, apoptosis, and regulation of cancer immunology. A simplified version of the combined figures is available as a Supplementary Figure (Figure S1).

It is evident that the cell cycle is an important process in carcinogenesis and that its dysregulation is crucial for tumor growth. In recent years, inhibition of the cell cycle has become an attractive target in cancer treatment [105,106]. The impact of PIWI proteins on the cell cycle can be assigned to the regulation of many proteins on different stages of cell division. PIWIL1 and PIWIL2 were reported to be able to interact with and alter the expression of both cyclins and cyclin-dependent kinases (CDKs), key regulators of the cell cycle. It was shown that their activity is important in maintaining the correct cell cycle. Dysregulation of these interactions can lead to uncontrolled cell proliferation, genomic instability, and chromosomal instability, all of which are hallmarks of cancer [107,108,109]. Therefore, it is evident that their regulation is important in the progression of malignancy. In recent years, a number of studies have shown how CDKs can be promising therapeutic targets [110,111,112]. PIWIL1 and PIWIL2, with their ability to alter cyclins and CDK machinery, furthermore confirm their influence on cell cycle and oncogenic characteristics. However, a high degree of diversity in the observed impacts of PIWIs on the cell cycle implies possible differences in the mechanisms behind PIWIs’ associations with the cell cycle depending on the type of cancer. Knudsen et al. point to high heterogeneity in requirements for both CDK and cyclins in cancer types [113]. This observation might suggest a possible reason for the high number of possible mechanisms behind regulation of the cell cycle by PIWIs. Also worth noting is the ability of PIWI proteins to alter the process of apoptosis. Inhibition of anti-apoptotic proteins is considered to have therapeutic potential [114]. As PIWI proteins can regulate apoptosis through different pathways, their possible impact on the regulation of apoptosis is a potential therapeutic target. However, exact implications need further examination, as differences between cancer types can be seen.

## 4. Invasion, Migration and Metastasis

Invasion and migration of cancer cells are pivotal mechanisms in cancer progression and metastasis. Unfortunately, because of their complexity, inhibition of these pathways in clinical applications has shown limited efficiency in cancer treatment [115,116].

A number of studies have shown the impacts of PIWI proteins on invasion and migration; therefore, their importance needs to be brought to attention.

In endometrial cancer, PIWIL1 overexpression resulted in increased migration and invasiveness of tumor cells. Additionally, it was revealed that this protein was responsible for an Epithelial-Mesenchymal Transition (EMT)-like phenotype, which was associated with increased mesenchymal markers and suppression of E-cadherin. Furthermore, PIWIL1 increased expression of CD44 and ALDH1, and cells with knockdown of PIWIL1 showed a decrease in these markers [117]. Invasion and migration of glioma cells were altered after inhibition of PIWIL1, supposedly by reducing the expression of MMP-2 and MMP-9 [70]. Similarly, inhibition of PIWIL1 in K562 cells resulted in decreased activity and the expression of MMP-2 and MMP-9 [60]. Li et al. revealed how PIWIL1 can become a co-activator of APC/C within PDAC cells, priming Pinin proteolysis via the APC/C-Ub pathway. Pinin is a critical cell adhesion-related protein; consequently, this process results in strengthened metastatic properties of cancer cells [118]. The previously described increase in STMN1 expression, meditated by PIWIL1, can result in increased invasion, as shown by Transwell assay [71]. As described before, miR-154-5p targets PIWIL1′ 3′UTR. This phenomenon can alter not only the cell cycle but also migration and invasion, which could be reversed by overexpression of PIWIL1 [78]. Knockout of PIWIL1 in gastric cancer cells is associated with increased migration, invasion, and metastasis, both in vitro and in vivo. It can be connected to NMD of mRNAs related to molecules responsible for cell adhesion, which ultimately results in oncogenic development [66].

As described above, PIWIL2 can promote NME2 binding to the c-Myc promoter, increasing its expression, and subsequently upregulating RhoA, which can further change tumor cell invasion and migration, by modulating F-actin filaments [94]. PIWIL2 interacts with Siah2, activating this pathway, which in turn promotes tumor growth and metastasis. Blocking PIWIL2 expression or inhibiting the Siah2 pathway attenuates DEX-induced migration, and invasion of CRC cells. The findings also reveal that DEX promotes EMT, a process critical for cancer metastasis, by upregulating PIWIL2 [96]. Increased invasion and migration can be meditated by PIWIL2 through the MAPK pathway, subsequently increasing the expression of MMP-9 [85]. Yang et al. described the involvement of PIWIL2 in prostate cancer cells. PIWIL2-knockdown PC-3 cells had significantly weaker invasion and metastasis abilities. Expression of molecules such as vimentin, N-cadherin, Twist, and MMP-9 was observed to be downregulated [119]. Li et al. reported the importance of PIWIL2 in colorectal cancer cells. The authors observed that PIWIL2 inhibition resulted in suppressed invasion and migration of cells. This phenomenon could have been achieved through changes in the activity of MMP-9 [65]. Similarly, another study showed how PIWIL2-induced CSC-delivered exosomes can increase migration, proliferation, and invasion of fibroblasts in vitro. These changes might be the result of increased expression of MMP-2 and MMP-9 [120]. Inhibition of PAK1 phosphorylation of TBCB by PIWIL2 can lead to increased migration and invasion of cancer cells [93].

Inhibition of PIWIL3 suppressed migration, invasion, and volume of the tumor. The authors reported altered expression of a number of cancer metastasis-related genes, such as MTA1, MMP-2, MMP-9, and RhoC. Furthermore, it was revealed that PIWIL3 might have an impact on the JAK2/STAT3 signaling pathway [100].

Another piece of research showed estrogen-induced upregulation of PIWIL4. Knockdown of PIWIL4 revealed its connection to the migration and invasion of breast cancer cells. It was suggested that this change in motility could be attributed to changes in expression of vimentin and N-cadherin [39]. As previously described, miR-154-5p can target PIWIL1 and suppress its activity in U251 and LN229 cells, resulting not only in an altered cell cycle but also suppression of invasion and migration abilities [77]. ECM shapes the tumor microenvironment, allowing cancer cells to invade and migrate to surrounding tissues, opening routes to metastasis [121]. MMP is a group of enzymes that have the ability to control ECM and are associated with cancer metastasis [122]. PIWI proteins are able to control MMPs, thereby regulating invasion, migration, and metastasis of cancer. However, MMPs are not the only proteins involved in this that are controlled by PIWI. This suggests a multidirectional impact of PIWIs on pathways involved in the metastatic properties of tumor cells.

## 5. Stem Cells

CSCs are increasingly recognized for their role in cancer treatment resistance and disease progression [123]. CSC is a subtype of cancer cell that is associated with relapse and tumor initiation. Characteristics of CSCs include their ability to self-renew and maintain stemness [124,125]. Tumor microenvironment and a number of different pathways are involved in the biology of CSCs, and better understanding of their influence is necessary for CSC-based targeted therapy [126].

Litwin et al. observed a correlation between the level of expression of PIWIL1 mRNA and cancer stem cell marker—OCT4 [127]. Huang et al. observed overexpression of PIWIL1 in 88% of glioblastoma specimens, with additional correlation with SOX2 [67]. Wang et al. revealed the involvement of PIWIL1 in regulation of genes such as *NANOG*, *OCT4*, and *SOX2* [76].

It has been shown that cells with CSC properties can be induced by reprogramming with PIWIL2. This process can result in cells that express mRNA of the stem cell-associated genes *OCT4*, *NANOG*, and *SOX2*. Additionally, it has been reported that these PIWIL2-GFP fibroblasts showed expression of three germ layer gene markers. These results showed the potential of PIWIL2 to be a factor associated with carcinogenesis and a novel approach to studying these mechanisms [128]. Two other research groups achieved similar results with this method. Shahali et al. obtained MEF-PIWIL2 cells, which were shown to be immune to hypoxic conditions [129]. Feng et al. observed that PIWIL2 could reprogram somatic cells into tumor-initiating cells. It was shown that the Wnt/β-catenin pathway plays an important role in maintaining the stem cell characteristics of TICs, which were reprogrammed by PIWIL2. This maintenance of TICs was supposedly mediated by β-catenin/CBP-mediated transcription of c-Myc, NANOG, OCT4, SOX2, and KLF4. Overall, it shows the importance of the role PIWIL2 plays in the carcinogenesis of cervical cancer [130]. Furthermore, it was reported that PIWIL2 could increase the expression of pluripotency genes, such as CD133, CD24, and SOX2 [88]. PIWIL2-overexpressing cells HaCaT exhibit increased levels of CSC markers: c-Myc, KLF4, NANOG, OCT4, and SOX2 [98]. The studies mentioned indicate that PIWI proteins can impact the stemness of cancer cells, suggesting their possible potential as therapeutic target. Worth noting is CSC’s abilities to influence tumor microenvironments and the metastatic properties of cells [131,132]. CSCs are also associated with processes like the cell cycle and chemoresistance [133,134,135]. This suggests that PIWIs might connect different pathways and different mechanisms of carcinogenesis.

## 6. Other Means of Influence on Cancer Cells by PIWI Proteins

### 6.1. Chemoresistance

In ovarian cancer cells, cis-platin resistance has been suggested to be connected to the expression of PIWIL2. It can be attributed to changes in chromatin modifications [136]. Wang et al. discovered the increased sensitivity of K562 cells to daunomycin after PIWIL1 overexpression [74]. Furthermore, PIWIL1 is involved in the chemoresistance of multiple myeloma. Inhibition of this protein resulted in increased vulnerability of MM cells to doxorubicin, bortezomib, and dexamethasone [76].

### 6.2. Tumor Microenvironment

PIWIL1 might be responsible for changes in cell metabolism and cell immune response in hepatocellular carcinoma. Overexpression of PIWIL1 resulted in accelerated fatty acid metabolism and higher secretion of the C3 component, which subsequently could result in P38 MAPK-mediated production of IL-10 in myeloid-derived suppressor cells in the tumor microenvironment [137]. PIWIL1 was also shown to be involved in angiogenesis. Li et al. observed a correlation between the expression of PIWIL1 and Ang-2 in breast cancer and uterine cervical cancer, and between PIWIL1 and both Ang-2 and Tie-2 in ovarian cancer [138]. Interestingly, PIWIL4 is able to inhibit the expression of MHC class II genes [53].

### 6.3. Circadian Rhythms

Tan et al. reported the possible role of PIWIL1 in altering circadian rhythms in cancer cells through two mechanisms. PIWIL1 can activate the PI3K-AKT signaling pathway by promoting interaction between SRC and PI3K, which results in phosphorylation and inactivation of GSK3β and eventually phosphorylation and ubiquitination of CLOCK and BMAL1 by GSK3β. The second way of suppressing circadian rhythms happens through the binding of PIWIL1 to the E-BOX region together with the CLOCK/BMAL1 complex, repressing transcription and resulting in the suppression of circadian rhythm [139]. A similar mechanism is mentioned in terms of PIWIL2 [140].

## 7. Concluding Remarks and Future Perspectives

PIWI proteins are involved in carcinogenesis through different mechanisms. Cell cycle dynamics, rate of proliferation, and induction of apoptosis can be altered through changes in expression of PIWI proteins. Similarly, migration, invasion, and metastatic properties are regulated by PIWIs. These changes might be connected with the ability of PIWIs to induce and control stemness in cancer cells. This is particularly evident in terms of PIWIL2. Whether CSC properties induced by PIWIs can promote other changes in cells such as cell cycle, proliferation, apoptosis, and metastatic characteristics remains poorly understood. However, it is necessary to outline differences in terms of effects caused by PIWIs in various types of cancer. Nevertheless, it is apparent that the cancer cell stemness induced by PIWIs can be a crucial factor, governing their ability to promote metastasis, chemoresistance, and even possible relapse of disease. One of the most prominent pathways engaged in PIWI-mediated tumorigenesis appears to be STAT3, PI3K, and MAPK. Regulation of this pathway by PIWI proteins can have an important influence on cancer cells, with STAT3 being particularly interesting, considering its diverse effects. However, other mechanisms, such as DNA methylation and chromatin modifications, are also mentioned. The connections between LC3, p62, and the mTOR pathway suggest that PIWIL1 may influence additional autophagic processes and protein degradation pathways. Together with PIWIL1’s interactions with PINK1/PARKIN, this indicates that the activation of these molecules may regulate other pathways involved in oxidative stress responses, such as the NRF2 pathway responsible for cellular protection against oxidative stress. This connection may serve as a target for future studies on the cellular effects induced by elevated PIWIL1 expression in cancer cells. The PIWIL1/DNMT1/PTEN signaling axis, involved in the progression of type I endometrial cancer, suggests a potential role for PIWIL1 in the PI3K/AKT signaling pathway, given that PTEN is the primary inhibitor of this pathway, which regulates cell growth, survival, and proliferation. PI3K/AKT is known to interact with the mTOR pathway, further linking this pathway to autophagy and apoptosis regulation, with PIWIL1 interactions with LC3 and p62 observed in MM cells. Dysregulation of PI3K/AKT may also impact other pathways, such as the FOXO pathway, which is associated with cell cycle regulation and stress response. The potential associations listed may be targets for future research on the role of PIWIL1 in carcinogenesis. To date, no direct associations between PIWIL2 expression and angiogenesis have been demonstrated. However, given the role of PIWIL2 in the PIWIL2/Siah2/HIF1α signaling axis, which promotes tumor growth through mechanisms related to cellular hypoxia response, an impact on the angiogenesis pathway is probable. Activation of HIF1α increases VEGF expression, suggesting possible involvement of PIWIL2 in angiogenic processes, setting a potential target for future research. PIWIL4 is associated with processes such as cell cycle arrest, apoptosis, cell migration, proliferation, and TGF-β signaling. By interplay with the TGF-β, MAPK/ERK, and FGF pathways, PIWIL4 may be involved in the EMT pathway. Associations between PIWIL4 and transcription factors such as Snail, Slug, Twist, and ZEB1, which promote EMT, have not yet been investigated. Current research indicates the possibility of such interactions between the expression of PIWIL4 and EMT-related proteins, which may be a tempting topic for future studies on the role of PIWIL4 in metastatic mechanisms in cancer. Exact uses and their potential efficiency need to be further examined, with special consideration of the impact on CSCs. Moreover, differences between cancer types need further examination to better classify the possible implementation of PIWI-targeted therapy in cancer. Another aspect necessary to explore is the possible adverse effects of such therapy. PIWI proteins are unquestionably an interesting topic in cancer biology; however, further research is needed to examine the possible interplay between different pathways regulated by PIWIs and their different functions depending on cancer type.

## Supplementary Materials

The following supporting information can be downloaded at: https://www.mdpi.com/article/10.3390/ijms252212217/s1.
