# Supplementary figures and images for: Mechanisms Behind the Impact of PIWI Proteins on Cancer Cells: Literature Review
Source: Int J Mol Sci. 2024 Nov 14;25(22):12217. doi: 10.3390/ijms252212217 (PMC11594409; doi:10.3390/ijms252212217)

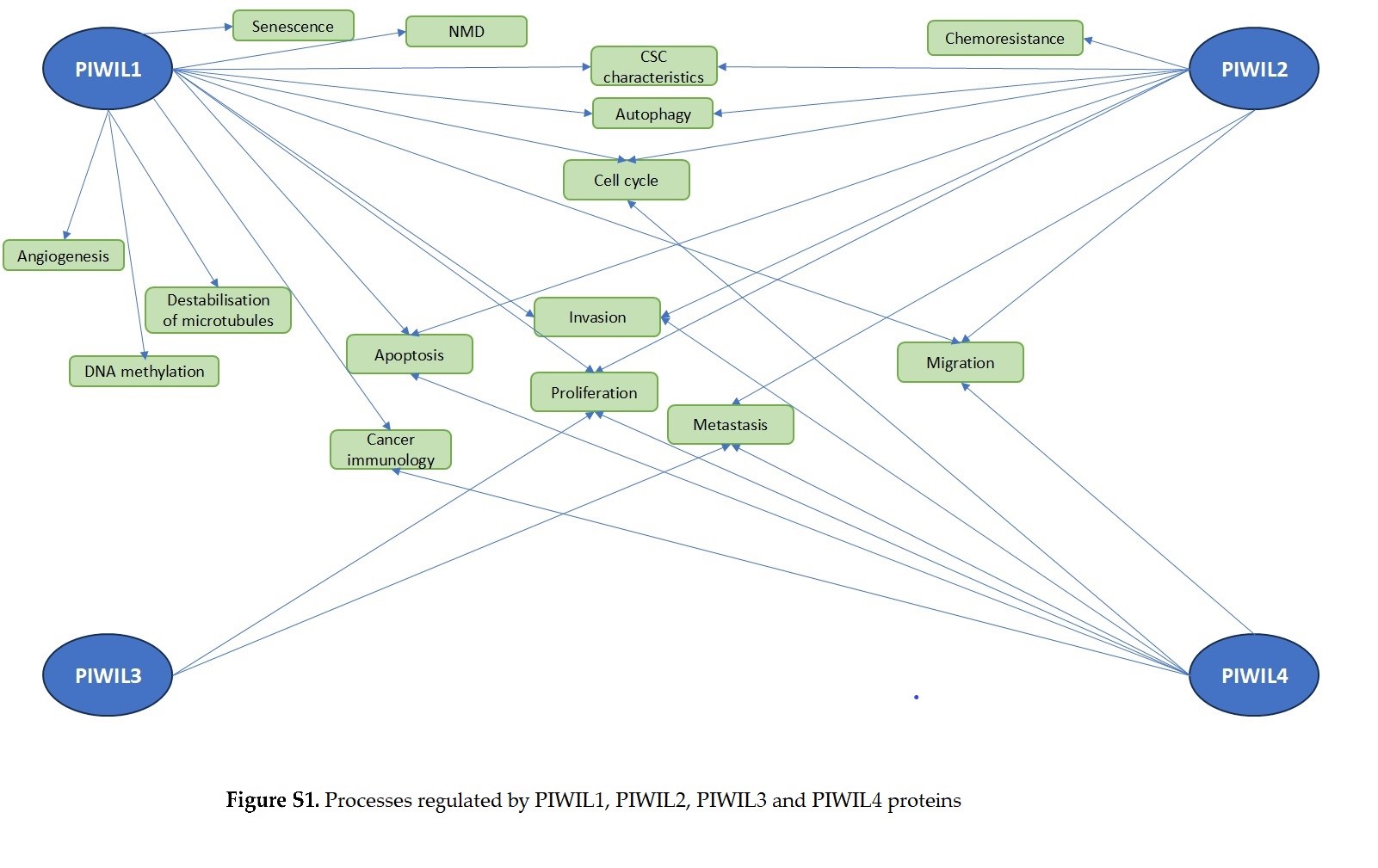

Supplement: Supplementary file 1 [file ijms-25-12217-s001.zip › ijms-3263179-supplementary.jpg]
